# The flavonoid isoliquiritigenin promotes lifespan extension of *Caenorhabditis elegans* through the transcription factor DAF-16 and aquaporin-2

**DOI:** 10.1590/1414-431X2025e14996

**Published:** 2026-08-07

**Authors:** Xuan Zhao, Limei Ren, Dandan Gu, Yasen Yao, Shuo Wang, Jiali Gao, Yonghao Qi

**Affiliations:** 1 Department of Bioengineering, School of Chemical Engineering, Shijiazhuang University, Shijiazhuang, Hebei, China; 2 Hebei Biopharmaceutical International Joint Research Center, Shijiazhuang University, Shijiazhuang, Hebei, China; 3 Department of Pathology and Key Laboratory of Kidney, Hebei Medical University, Shijiazhuang, Hebei, China; 4 Key Laboratory of Innovative Drug Research and Evaluation in Hebei Province, Hebei Medical University, Shijiazhuang, Hebei, China

**Keywords:** Lifespan, Antioxidant capacity, Membrane water permeability, DAF-16, AQP-2

## Abstract

Aging is associated with the accumulation of oxidative damage. Isoliquiritigenin (ISL), a natural flavonoid, exhibits antioxidant properties, but its effect on lifespan and the underlying mechanisms remains incompletely understood. This study aimed to investigate whether ISL extends lifespan in *Caenorhabditis elegans* through the transcription factor DAF-16 and the aquaporin AQP-2. We employed lifespan assays, stress resistance tests, intracellular ROS measurement, osmotic water permeability assays in *Xenopus* oocytes, genetic manipulation (using mutants and RNAi), quantitative real-time PCR, and fluorescence microscopy to assess DAF-16 localization and target gene expression. ISL extended the mean lifespan of wild-type *C. elegans* by 15.35% at 20 μM (P<0.01). It enhanced resistance to oxidative stress (36.54% higher survival under paraquat) and heat shock (20.56% higher survival at 37°C), and reduced intracellular ROS levels. Mechanistically, ISL increased DAF-16 nuclear translocation by 44.3% (P=0.003), and upregulated the expression of its target genes, including *sod-3* (3.2-fold, P=0.008) and *aqp-2* (2.9-fold, P=0.012). ISL also increased the osmotic water permeability of *Xenopus* oocytes by 1.7-fold (P=0.007), and the effect was abolished by the aquaporin inhibitor HgCl_2_. Genetic ablation of *aqp-2* nullified ISL-induced lifespan extension (P=0.65 *vs* control) and ROS reduction. Crucially, *aqp-2* RNAi suppressed ISL-driven DAF-16 nuclear accumulation and *sod-3* expression, establishing a feedforward loop. ISL extended lifespan and enhanced stress resistance in *C. elegans* by activating a DAF-16/AQP-2 regulatory module, thereby linking water homeostasis to the transcriptional control of aging.

## Introduction

Excessive oxidative damage caused by sunlight exposure and air pollution tends to accelerate the aging process (1,2). Isoliquiritigenin (ISL), a flavonoid derived from the roots and rhizomes of licorice (*Glycyrrhiza* species), has been shown to enhance redox regulation and exhibit anti-tumor and anti-inflammatory capacities in animals. Free radicals may mediate at least some of the effects of ISL intake on human health. Regarding its pro-oxidant potential, ISL inhibits tumor growth through reactive oxygen species (ROS)-mediated regulation of the Jak2/STAT3 pathway (3). Conversely, ISL can also reduce ROS levels to protect cells from oxidative injuries during inflammation (4). Due to the prominent antioxidant properties of ISL, several studies have suggested a protective effect of ISL against oxidative stress-related diseases, such as cardiomyocyte contractile dysfunction and attenuation of hepatic oxidative damage (5,6). However, whether ISL extends lifespan remains unclear.

In animals, one hallmark of aging is decreased cellular water content, resulting in the accumulation of age pigments, oxidative damage, and reduced metabolic capacity. The water content of human cells can be suppressed by oxidative signals (7). Due to its antioxidant properties, ISL may increase cellular water content and the lifespan of *Caenorhabditis elegans* (*C. elegans*). The mechanisms might involve the water channel aquaporin (AQP)-2 protein (homologous to mammalian AQP-3). In mammals, AQP-3 acts as a biomarker of aging-induced skin alterations (8). Mammalian AQP-3 also mediates H_2_O_2_ uptake and regulates immune responses as well as tumor cell migration. However, whether and how worm AQP-2-mediated water permeability increases antioxidant capacity and lifespan requires further research. Possible molecular mechanisms may involve the DAF-16/FOXO transcription factor.

In *C. elegans*, FOXO transcription factor DAF-16 contributes to the longevity of wild-type worms cultured on bacteria under standard culture conditions. Suppressing *daf-16* gene expression accelerates the rate of worm aging and shortens lifespan. In humans, FOXO may mediate some of the effects of oxidative stress on lifespan (9). In response to oxidative stress, FOXO rapidly accumulates in the nucleus, where it promptly stimulates the transcription of downstream longevity genes (10). This rapid translocation of DAF-16/FOXO and the subsequent change in gene expression are thought to enhance stress resistance, ultimately extending lifespan. Although the connection between DAF-16/FOXO and aging is well-established, the impact of the *C*. *elegans* water channel AQP-2 (a homolog of human AQP-3) on DAF-16/FOXO and lifespan remains poorly understood.

The role of aquaporins in aging appears to be an evolutionarily conserved mechanism. In mammals, FOXO transcription factors stimulate the expression of homologous aquaporins under low insulin conditions (11). Furthermore, genetic depletion of specific aquaporins in mammalian models can lead to metabolic disorders and a shortened lifespan, underscoring the critical importance of these channels in regulating metabolism and aging (12,13).

In this study, we hypothesized that ISL extends the lifespan of *C. elegans* by enhancing antioxidant defense and cellular water homeostasis through mechanisms involving DAF-16 and AQP-2. The nematode *C. elegans* was employed as a model organism due to its highly conserved signaling pathways.

## Material and Methods

### Animals

Adult female *Xenopus laevis* frogs weighing 150±10 g were purchased from the Institute of Developmental Biology, Chinese Academy of Sciences. The frogs were fed frog chow twice a week and housed in plexiglass tanks containing carbon-filtered water. This study was approved by the Ethics Committee of Shijiazhuang University (Approval No: [IACUC-SJZU-2024-10002]).

### Isolation of *Xenopus* oocytes

Stage V-VI *Xenopus* oocytes were harvested using well-established procedures (14). Briefly, frogs were anesthetized by immersion in a 0.2% tricaine (3-aminobenzoic acid ethyl ester) solution for 6 min and then placed on ice for hypothermia. A 1-cm incision was made in the abdominal wall to expose and remove one lobe of the ovary. The ovarian tissue was rinsed several times with Ca^2^-free Modified Barths' saline (MBS) until the solution became clear, followed by digestion with gentle agitation for 35-45 min in approximately 20 mL of sterile-filtered Ca^2^-free MBS containing 2.35 mM collagenase (type IA). The released oocytes were then rinsed three times with Ca^2^-free MBS, manually sorted, and stored in MBS at 18°C.

### Strains and culture condition

The worm strains Bristol N_2_ (wild type), CF1038 *daf-16*(mu86), TJ356 zIs356 [*daf-16*:GFP], RB1715 aqp-2 (ok2159), and CF1553 muIs84 [pAD76(*sod-3*::GFP)] were used. All strains in this study were obtained from the Caenorhabditis Genetics Center (CGC, University of Minnesota, USA). Nematodes were cultured using standard methods. Worms were cultured on nematode growth medium (NGM) agar plates at 20°C seeded with *Escherichia coli* OP50.

### Chemicals and solutions

ISL, a naturally occurring chalcone-type flavonoid, features a distinct chemical architecture comprising two aromatic rings (rings A and B) connected by a three-carbon α,β-unsaturated carbonyl bridge. Ring A is a resorcinol-type moiety with hydroxyl groups at the C2' and C4' positions, whereas ring B is a 4-hydroxyphenyl unit. This specific 2',4',4-trihydroxychalcone configuration, characterized by its conjugated ketone system, is fundamental to ISL’s electron-donating capacity and redox-modulating properties. The integrity of this structure is crucial for the diverse pharmacological activities, including potent antioxidant, anti-inflammatory, and anticancer effects, which are underpinned by ISL’s ability to interact with multiple biological targets. For this experiment, ISL was obtained commercially from the Shanghai Institute of Materia Medica, China, with a certified purity of 98%.

ISL was dissolved in DMSO (Sigma-Aldrich, USA) and was added directly to OP50 to a final concentration of 0, 10, 20, and 40 mM. All groups contained 0.01% (v/v) DMSO as the solvent control. *Escherichia coli* expressing dsRNA corresponding to gene *aqp-2* was purchased from Suzhou GenePharma Co., Ltd., China. MBS, tricaine, and collagenase (type IA) were purchased from Sigma-Aldrich (USA). Fluorescence probe H2DCF-DA (2′,7′-dichlorofluorescein diacetate) was obtained from Beyotime Biotechnology Co., Ltd., China. RNA prep Pure Kit was obtained from Tiangen Biotechnology Co., Ltd., China.

### Lifespan and stress resistance assay

The wild-type *C. elegans* strain N2 was used for the lifespan assay. Synchronized late-L4 larvae were transferred to NGM plates seeded with OP50 *E. coli* and supplemented with the indicated concentrations of ISL (0, 10, 20, and 40 mM). Survival was scored daily (15). The effect of ISL on heat shock stress of worms was examined on day 3 of the lifespan assay. Worms were treated with acute heat stress at 37°C, and survival was monitored each hour. The effect of ISL on oxidative stress resistance was examined on day 3. Worms were transferred to a new NGM containing paraquat (30 mM), and survival was monitored at 24, 48, 72, 96, and 120 h (6). In both stress resistance assays, paralyzed nematodes were considered dead. All NGM plates contained 50 μM 5-fluoro-2'-deoxyuridine (5-FU) to prevent progeny development in all assays. Each assay was performed in triplicate.

### RNA interference assay

Gravid adult worms of the *daf-16*::*gfp* and *sod-3*::*gfp* strains were transferred to plates seeded with *E. coli* expressing dsRNA specific to the *aqp-2* gene and allowed to lay eggs for 2-3 days at 22°C. After removing the adults, the eggs were incubated for 40 h to develop into L4 larvae. These L4 larvae (*daf-16*::*gfp*; *aqp-2*(RNAi) and *sod-3*::*gfp*; *aqp-2*(RNAi)) were then used for experiments (16).

### Intracellular ROS assay

Intracellular ROS were examined using H_2_DCF-DA (2′,7′-dichlorofluorescein diacetate), a fluorescence probe. Synchronized L4 larvae were cultured on NGM plates with or without ISL until day 3 of adulthood. The fluorescence intensity was then measured at 37°C via DCF fluorescence (excitation wavelength: 485 nm; emission wavelength: 535 nm). This assay was performed three times.

Osmotic water permeability (Pf) was determined according to previous methods (17). *Xenopus* oocytes were incubated for three days at 18°C in 200 mOsM MBS with or without 20 μM ISL. Osmotic water swelling was performed at 22°C by transferring oocytes from 200 mOsM (OsM_in_) into 70 mOsM (OsM_out_) MBS diluted with deionized water. The longest (D1) and shortest (D2) diameters of the oocytes were measured at 30 s intervals until the oocyte membrane ruptured. Oocyte volumes (V) at each time point were calculated relative to the initial observed volume (V_0_) as follows: V_0_ = 9×10^-10^ m^3^; the initial oocyte surface area (S) = 4.5×10^-6^ m^2^; the molar volume of water (V_w_) = 1.8 × 10^-5^ m^3^ mol^-1^ × 1); V/V_0_ = (D1 × D2)^3/2^ / (Dl_0_ × D2_0_)^3/2^ × 2); Pf = [V_0_ × d(V/V_0_) / dt] / [S × Vw × (OsM_in_ - OsM_out_)].

### DAF-16 sub-cellular distribution

TJ356 (*daf-16*::*gfp*) was pretreated with or without ISL for three days from the L4 stage. Worms were transferred to a glass slide covered with 2% agarose. Image acquisition was performed with fluorescence microscopy (Nikon Eclipse 80i, Japan) at 200× magnification. Subcellular localization of DAF-16::GFP was determined as previously described (18). Worms with at least twenty green fluorescence dots were scored as nuclear, and worms with five or fewer green fluorescence dots were scored as cytosolic. The others were categorized as intermediate. Thirty worms were monitored in each trial, and this assay was repeated three times.

### Quantitative real-time PCR

Synchronized L4 worms were treated with or without ISL for three days. Total RNA was extracted using RNA Prep Pure Kits and reverse-transcribed into cDNA with Quantscript RT Kits. For the qPCR assay, approximately 200 ng of cDNA was used per reaction. The reactions were carried out using SKAPA SYBR^®^ 2×qPCR Master Mix (USA) as the detection method and performed on the Light Cycler (Roche, Switzerland) platform. Relative fold changes in gene expression were calculated using the 2^−ΔΔCt^ method, with act-1 as the internal control (19).

The following primers were used for RT-PCR: *aqp-2*: Forward: 5′-ATACCCAGTCAACCCAGCTC-3′; Reverse: 5′-TCCTGGTTTCCGGTGAGTAC-3′. *sod-3*: Forward: 5′-CGAGCTCGAACCTGTAATCAGCCATG-3′; Reverse: 5′-GGGGTACCGCTGATATTCTTCCAGTTG-3′. *act-1*: Forward: 5′-CCAGGAATTGCTGATCGTATGCAGAA-3′; Reverse: 5′-TGGAGAGGGAAGCGAGGATAGA-3.

### Fluorescence quantification of sod-3::GFP

The s*od-3*::*gfp* reporter strain CF1553 was used. Worms were treated with or without ISL from the L4 stage for 48 h, and then 100 worms were transferred onto a 96-well plate with 100 μL of S buffer (100 mM NaCl, 50 mM potassium phosphate, pH 6.0) per well. The GFP fluorescence intensity was measured using a Tecan Infinite 200 PRO (excitation wavelength: 485 nm; emission wavelength: 530 nm; Tecan, Switzerland) (20). This assay was repeated three times.

### Statistical analysis

All lifespan data are reported as means±SE and were analyzed by Kaplan-Meier survival method. SPSS 18.0 (IBM, USA) or Origin 2019 softwares were used for statistical calculation. Significant differences between the experimental and control groups were analyzed by the log-rank test, one-way ANOVA, or Student's *t*-test. Differences with P<0.05 and P<0.01 were considered statistically significant.

## Results

### ISL extended the lifespan and antioxidant capacity of *C. elegans*

**Figure 1:**
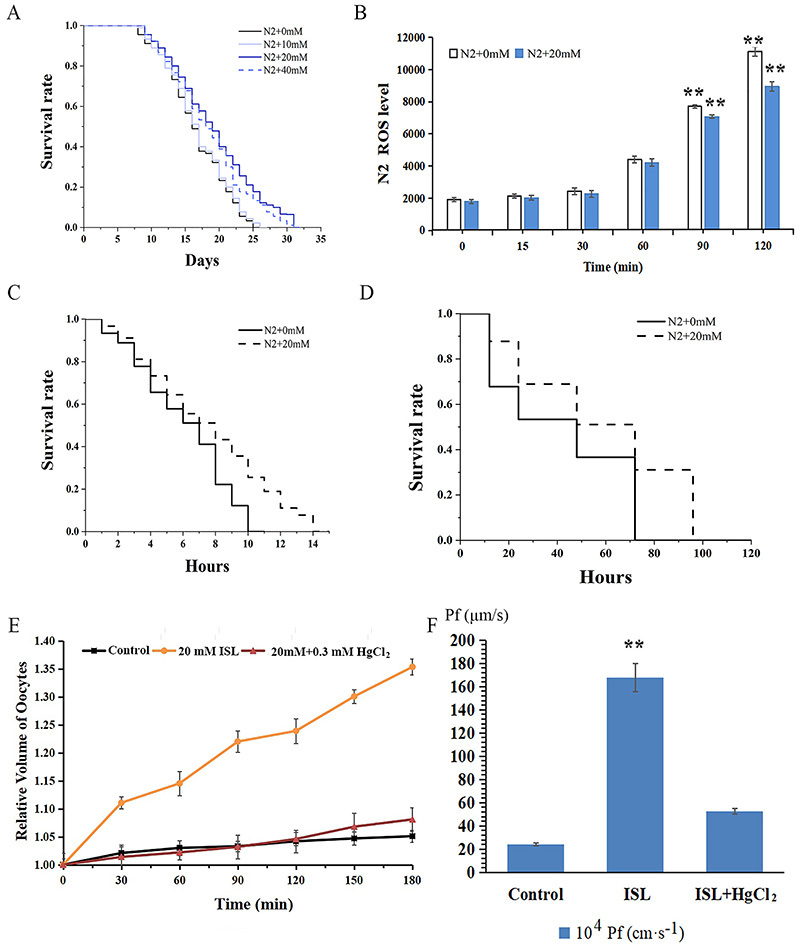
Isoliquiritigenin (ISL) extends lifespan, enhances antioxidant capacity in *C. elegans*, and increases osmotic water permeability in oocytes at the optimal dose. **A**, Survival curves of wild-type worms fed with 0, 10, 20, or 40 mM ISL. Statistical details are provided in Table 1 (n>200). **B**, Intracellular reactive oxygen species (ROS) levels in wild-type (N2) worms. Data are reported as means±SD (**P<0.01; Student's *t*-test). **C**, Survival rates of 3-day-old adult N2 worms under acute heat stress at 37°C. Viability was scored hourly. **D**, Survival rates of 3-day-old adult N2 worms under paraquat-induced oxidative stress. Viability was monitored at the indicated time points. Statistical details are provided in Table 2 (n>200). **E**, Time course of relative volume changes in oocytes transferred from 200 mOsm to 70 mOsm MBS. Data are reported as means±SD (n=15). **F**, Osmotic water permeability (Pf) of oocytes. Data are reported as means±SD (n=15) (**P<0.01; Student's *t*-test)

To investigate the effect of ISL on the lifespan of *C. elegans*, wild-type worms were fed *E. coli* OP50 on NGM plates supplemented with 0, 10, 20, 40 mM ISL. At the optimal concentration of 20 mM, ISL extended the mean lifespan by approximately 15.35% (Figure 1A and Table 1).

**Table 1:** Lifespan of wild-type *C. elegans* (N_2_) with different doses of isoliquiritigenin (ISL) treatment.

Strain	ISL dose	Lifespan (days)	Extended (%)	P-value
N2	0 mM	17.52±0.12		
N2	10 mM	17.83±0.17	1.77%	<0.05
N2	20 mM	20.21±0.43	15.35%	<0.05
N2	40 mM	19.43±0.38	10.9%	<0.001

Data are reported as mean±SE. Log-rank test and Kaplan-Meier survival analysis.

To determine whether this lifespan extension was mediated by enhanced antioxidant capacity, we examined the survival of wild-type (N2) worms under oxidative stress and heat shock stress conditions by 36.54 and 20.56%, respectively (Figure 1C and D, and Table 2). Consistent with these results, ISL treatment effectively reduced intracellular ROS levels in wild type worms (Figure 1B). Thus, the observed enhancement in stress resistance coupled with the reduction in ROS levels suggests that ISL extends the lifespan of *C. elegans* by boosting its antioxidant capacity.

**Table 2:** Lifespan of wild-type *C. elegans* (N_2_) worms under stress condition.

Strain	Stress	ISL dose	Lifespan (days)	Extended (%)	P-value
N_2_	Heat shock stress	0 mM	7.10±0.03		
		20 mM	8.56±0.07	20.56%	<0.01
N_2_	Oxidant stress	0 mM	59.47±0.13		
		20 mM	81.20±0.08	36.54%	<0.01

Data are reported as mean±SE. Log-rank test and Kaplan-Meier survival analysis.

### ISL elevated the Pf of *Xenopus* oocytes

To determine whether ISL affected the Pf of cells, we tested its effect on swelling and Pf in *Xenopus* oocytes. After 72 h of ISL incubation, oocytes demonstrated more rapid osmotically driven increases in relative volume after transfer from 200 to 70 mOsM MBS (Figure 1E), suggesting that ISL promotes osmotic swelling. Regarding Pf, the oocytes incubated with ISL had higher Pf than those incubated without ISL (Figure 1F).

Subsequently, we inhibited the water channel protein in oocytes with HgCl_2_. Results showed that inhibiting AQPs suppressed the effect of ISL on volume swelling and Pf (Figure 1F). Together, these findings revealed that ISL elevates the osmotic water permeability via AQPs.

### Aqp-2 was required for ISL's longevity and antioxidant effects

**Figure 2:**
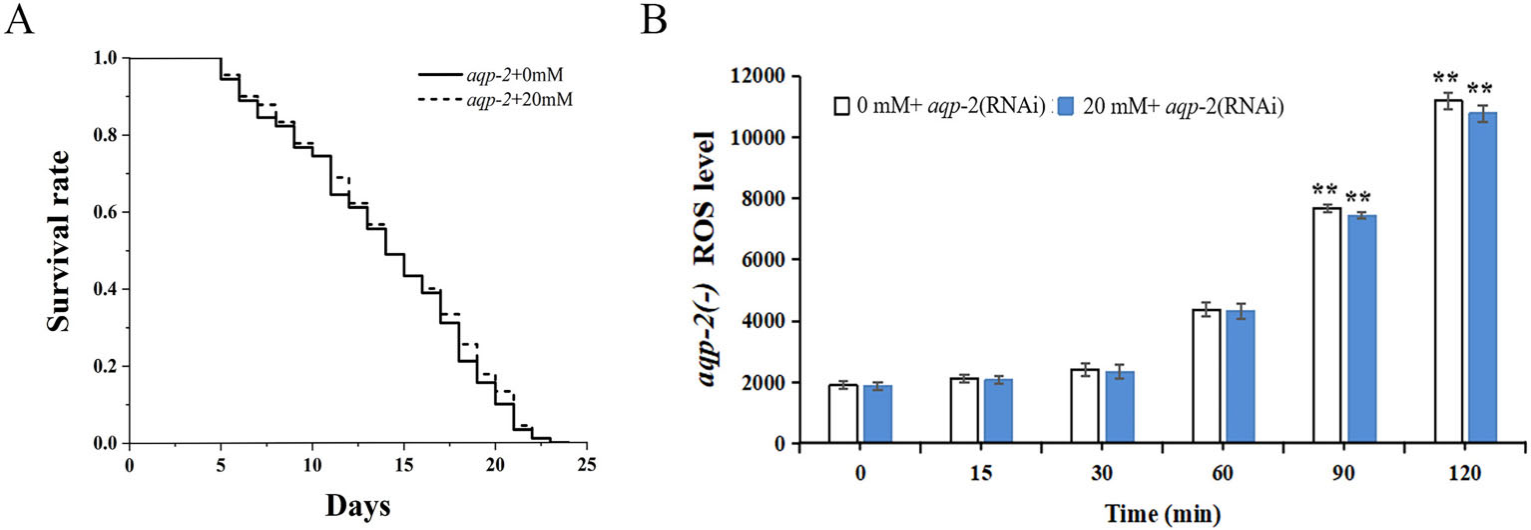
Aquaporin (AQP)-2 is required for the lifespan-extending and antioxidant effects of isoliquiritigenin (ISL). **A**, Survival curves of *aqp-2*(-) mutants with or without ISL treatment. Statistical details are provided in Table 3 (n>200). **B**, Intracellular reactive oxygen species (ROS) levels in *aqp-2*(-) mutants. Data are reported as means±SD (**P<0.01; Student's *t*-test).

To determine whether *aqp-2* was involved in the ISL-induced lifespan effect, the lifespan and intracellular ROS level of *aqp-2*(-) mutants were assessed with and without ISL treatment. In contrast to its impact on wild-type worms, ISL feeding did not significantly affect the lifespan or intracellular ROS levels of these mutants (Figure 2A and B, and Table 3). The lack of significant lifespan extension in *aqp-2*(-) mutants (P=0.65 *vs* untreated control, Table 3) contrasted starkly with the significant extension observed in wild-type worms (P<0.01, Table 1), underscoring the essential role of *aqp-2*. This pronounced effect, observed in multiple tests, revealed that *aqp-2* is required for the longevity and antioxidant effects of ISL.

**Table 3:** Lifespan of *daf-16(-)* and *aqp-2(-) C. elegans* mutants with different doses of isoliquiritigenin (ISL) treatment.

Genotype	ISL dose	Lifespan (days)	Extended (%)	P-value
*daf-16(-)*	0 mM	15.10±0.13		
	20 mM	15.27±0.37	1.23%	<0.05
*aqp-2(-)*	0 mM	14.96±0.23		
	20 mM	15.24±0.28	1.87%	<0.05

Data are reported as mean±SE. Log-rank test and Kaplan-Meier survival analysis.

### ISL improved an *aqp-2*-dependent DAF-16 nuclear accumulation

**Figure 3:**
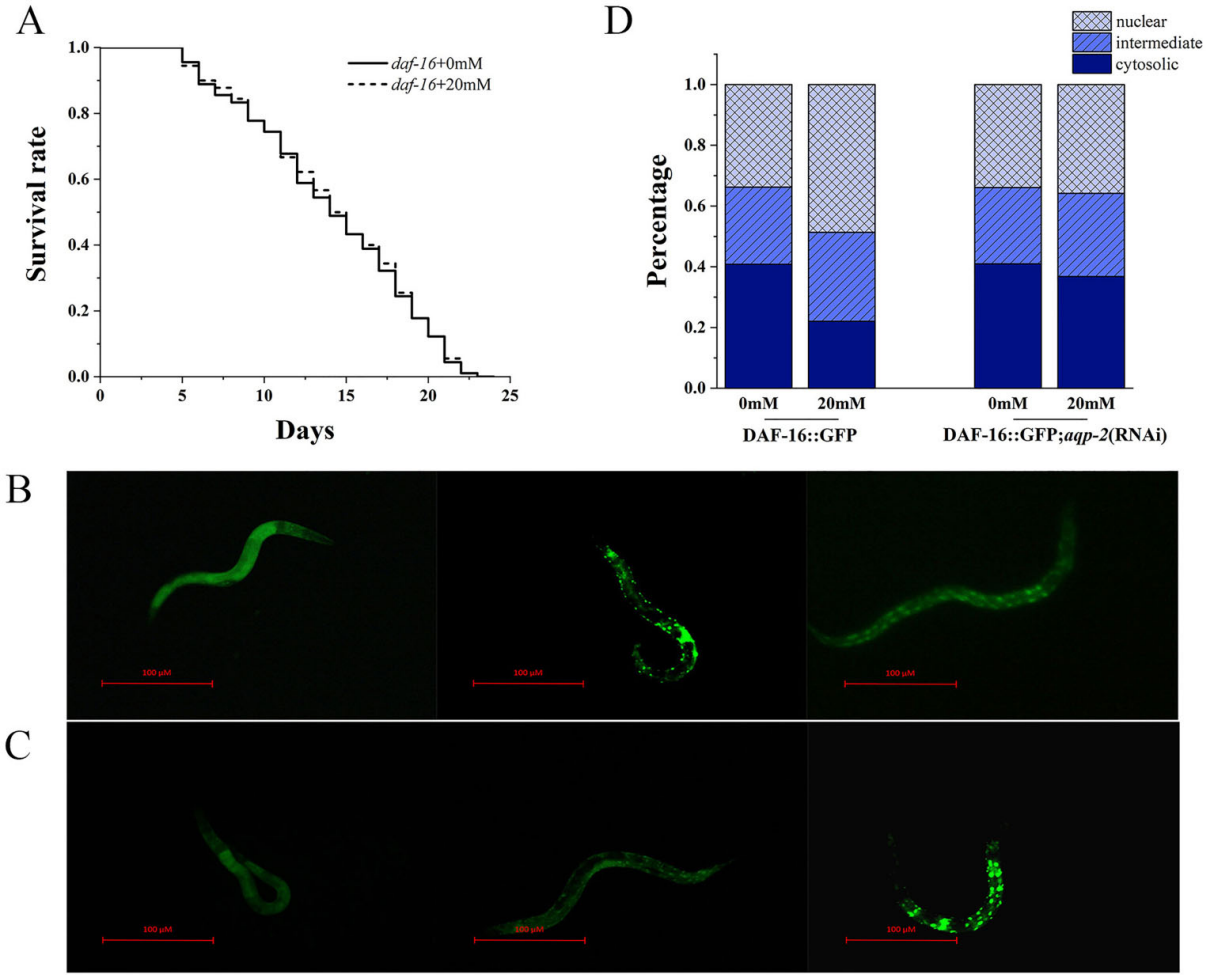
Isoliquiritigenin (ISL) promoted DAF-16 nuclear translocation in an aquaporin (AQP)-2-dependent manner. **A**, Survival curves of *daf-16*(-) mutants with or without ISL treatment. Statistical details are provided in Table 3 (n>200). **B**, Representative micrographs of TJ356 (*daf-16*::*gfp*) worms categorized as cytosolic, intermediate, or nuclear localization. Scale bar, 100 µm (200× magnification). **C**, Representative micrographs of TJ356 (*daf-16*::*gfp*) worms subjected to *aqp-2* RNAi, showing the categorized localization patterns. Scale bar, 100 µm (200× magnification). **D**, Quantitative analysis of DAF-16::GFP localization in the indicated strains following a 3-h treatment with or without 20 mM ISL. Worms were categorized as described in Methods. Data are from three independent experiments (n=90). One-way ANOVA was used for comparisons.

To test whether *daf-16* was involved in the ISL-induced lifespan extension, the lifespan of *daf-16*(-) mutants was examined. We found that ISL did not further extend the lifespan of *daf-16*(-) mutants (Figure 3A). In the DAF-16::GFP nuclear translocation assay, results showed that ISL increased DAF-16::GFP nuclear localization (control: 33.78%; ISL: 48.68%) (Figure 3B and D). Thus, these data revealed that ISL extended the lifespan of worms via DAF-16.

Because the antioxidant effect of ISL relies on *aqp-2* and the lifespan of the *aqp-2*(-) phenotype was similar to that of *daf-16*(-) mutants, we tested the possibility that AQP-2 might function within a regulatory pathway involving DAF-16. To verify this hypothesis, we tested the effect of ISL on DAF-16 nuclear translocation in *daf-16*::*gfp*;*aqp-2*(RNAi) worms. Results revealed that *aqp-2* knockdown suppressed DAF-16 nuclear accumulation under ISL treatment (Figure 3C and D). To summarize, ISL improved worms DAF-16 nuclear translocation to extend the lifespan of worms, which required *aqp-2*.

### ISL increased an *aqp-2*-dependent DAF-16 target gene expression

**Figure 4:**
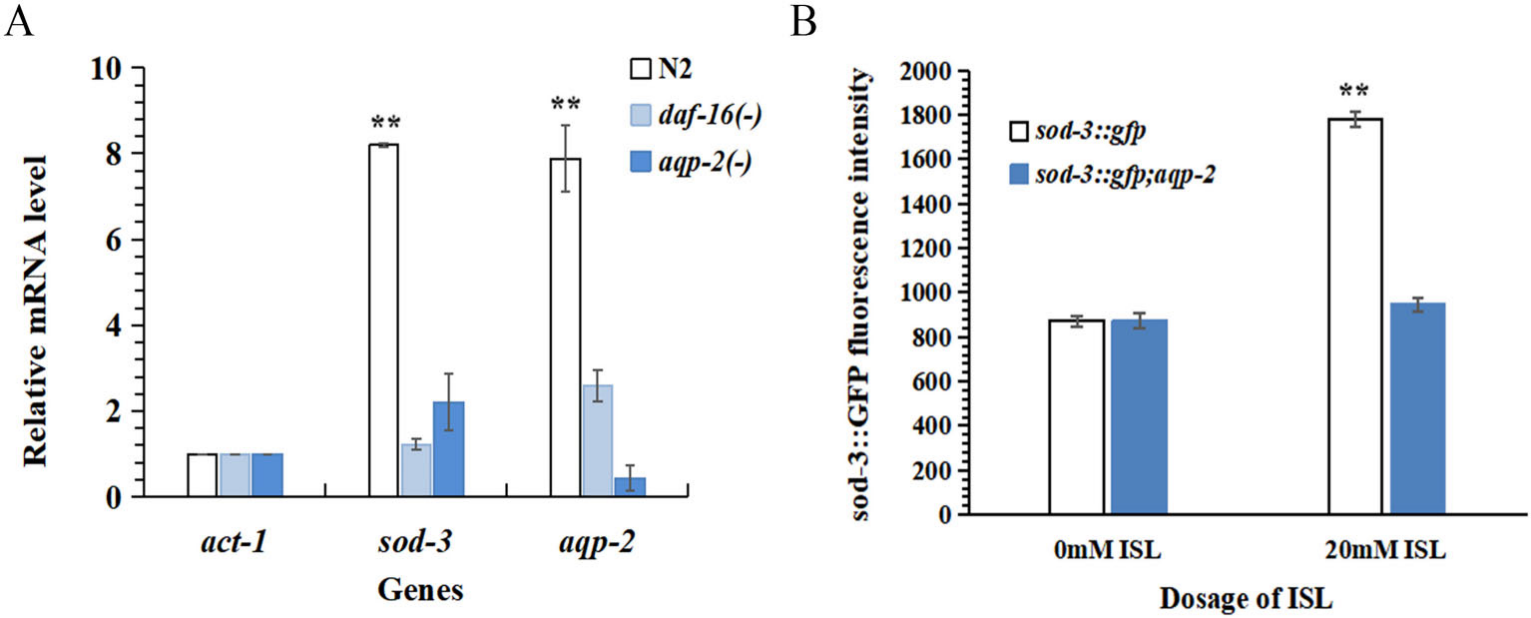
Isoliquiritigenin (ISL) upregulates the expression of DAF-16 target genes *sod-3* and *aqp-2* in a mutually dependent manner. **A**, mRNA levels of *sod-3* and *aqp-2* in wild-type *C. elegans*, *daf-16*(*-*) mutants, and *aqp-2*(-) mutants with or without 20 mM ISL treatment. Gene expression was analyzed by quantitative real-time PCR using *act-1* as an internal control. **B**, Quantification of GFP fluorescence intensity in *sod-3*::*gfp* and *sod-3*::*gfp*; *aqp-2*(RNAi) transgenic worms. Worms were treated with or without ISL for 48 h prior to measurement with a fluorescence microplate reader. Data are reported as means±SD (n=100 per group; **P<0.01; Student's *t*-test).

To determine whether the nuclear-accumulated DAF-16 upon ISL treatment stimulates the transcription of its target antioxidant genes, we examined the transcription and expression levels of *sod-3* in N2 and *sod-3*::*gfp* transgenic worms. As expected, transcription and expression of *sod-3* were increased in ISL-treated worms compared to untreated controls (Figure 4A and B). Interestingly, ISL treatment also improved the mRNA level of *aqp-2* in wild-type worms (Figure 4A). To verify whether *aqp-2* is also the target gene of DAF-16, we tested its transcription level of *aqp-2* in *daf-16*(-) mutants. As shown in Figure 4A, ISL did not improve *aqp-2* transcription in *daf-16*(-) mutants compared to the untreated mutant control. Thus, *aqp-2* is also the target gene of the transcription factor DAF-16.

Since *aqp-2* deletion suppressed DAF-16 nuclear accumulation (Figure 3D), we sought to verify whether *aqp-2* mutation would inhibit the effect of ISL on DAF-16 target gene expression. Results revealed that ISL did not elevate the *sod-3* transcription level in *aqp-2* loss-of-function mutants (Figure 4A), and *aqp-2* deletion inhibited the upregulation of *sod-3* expression by ISL (Figure 4B). To conclude, under ISL treatment, AQP-2 might also act as a feedback regulator of DAF-16.

## Discussion

Aging is driven by the accumulation of oxidative damage, a key contributor to age-related pathologies (21). Maintaining cellular hydration is vital, given the association between dehydration and increased morbidity in the elderly (6). Aquaporins, which include mammalian AQP-3 (homologous to *C. elegans* AQP-2), are implicated in aging and cellular hydration (22,23). Notably, AQP-3 expression is reduced in photoaged skin (24,25). Therefore, natural compounds that can enhance osmotic permeability and bolster antioxidant defenses hold significant promise for mitigating age-related physiological decline.

Our study demonstrated that the natural flavonoid ISL extended lifespan and enhanced antioxidant capacity in *C. elegans* through mechanisms that require both the transcription factor DAF-16 and the aquaporin AQP-2. At its optimal concentration of 20 µM, ISL increased the mean lifespan of wild-type worms by 15.35%. Furthermore, it improved survival under oxidative and heat stress and reduced intracellular ROS levels. The crucial role of DAF-16 was confirmed, as the lifespan extension was abolished in *daf-16*(-) mutants, consistent with its previously established function in longevity (26,27). Mechanistically, ISL promoted DAF-16 nuclear localization and upregulated its downstream antioxidant target, SOD-3.

Given the association between decreased cellular water content and aging (28), we investigated ISL's effect on water permeability. ISL increased the Pf and swelling in *Xenopus* oocytes, an effect that was blocked by the AQP inhibitor HgCl_2_, suggesting AQP involvement. Since AQP-2 is a key water channel in *C. elegans* (homologous to human AQP-3, which is vital for skin hydration (29)), we hypothesized it might mediate ISL's effects. Indeed, ISL failed to extend lifespan or reduce ROS in *aqp-2*(-) mutants, establishing AQP-2 as essential for ISL's benefits.

DAF-16 orchestrates longevity by regulating a network of target genes. We found that ISL upregulated the expression of both *sod-3* and *aqp-2*, with the induction of *aqp-2* being strictly DAF-16-dependent. This identifies *aqp-2* as a novel transcription target of DAF-16 in ISL-mediated longevity. Intriguingly, AQP-2 is not merely a passive target but participates in a feedforward loop, as its deletion impaired ISL-induced nuclear translocation of DAF-16 and the subsequent expression of *sod-3*. Although the precise molecular mechanism of this feedback requires further elucidation, our findings revealed a novel interplay between water homeostasis and transcriptional regulation in aging and defined a compelling model for future investigation.

The use of HgCl_2_ to inhibit AQPs, while common, is not entirely specific, as it can affect other proteins (30). However, its consistent effect in abolishing the ISL-induced Pf increase strongly supports the involvement of AQPs. The specific dependence on *aqp-2* in the worm model further strengthened this conclusion. The statistical comparisons for lifespan in Figure 2 and Table 3 show that ISL treatment did not significantly extend lifespan in *aqp-2*(-) mutants, unlike the significant extension seen in wild-type worms, confirming the essential role of *aqp-2*. The apparent discrepancy in basal DAF-16::GFP localization between results shown in Figure 3B-D is likely due to representative images *versus* quantitative data.

## Conclusion

In conclusion, ISL extended lifespan and enhanced oxidative stress resistance in *C. elegans* through mechanisms involving both systemic antioxidant regulation and aquaporin-mediated water homeostasis. Mechanistically, ISL induced nuclear translocation of DAF-16/FOXO, upregulating the expression of downstream targets *aqp-2* (an aquaglyceroporin critical for osmotic balance) and *sod-3* (a mitochondrial superoxide dismutase). This dual activation enhanced cellular antioxidant capacity and modulated water metabolism, collectively contributing to longevity. Intriguingly, we identified a feedback loop wherein AQP-2 reciprocally amplified DAF-16 activity, suggesting a self-reinforcing mechanism for ISL-mediated lifespan extension. The evolutionary conservation of this pathway is supported by evidence that FOXO transcription factors regulate homologous aquaporins in mammalian systems (30,31). These findings not only elucidated a novel DAF-16/AQP-2 axis in aging regulation but also proposed ISL as a potential dietary intervention to delay age-related physiological decline in higher organisms.

## Data Availability

The data that support the findings of this study are available from the corresponding author upon reasonable request.
